# Impact of Heavy Metals on Human Male Fertility—An Overview

**DOI:** 10.3390/antiox10091473

**Published:** 2021-09-15

**Authors:** Andrea López-Botella, Irene Velasco, Maribel Acién, Paula Sáez-Espinosa, José-Luis Todolí-Torró, Raquel Sánchez-Romero, María José Gómez-Torres

**Affiliations:** 1 Service of Obstetrics and Gynecology, Unit of Human Reproduction, FISABIO—San Juan University Hospital, Carretera Alicante-Valencia s/n, 03550 San Juan de Alicante, Spain; alb59@alu.ua.es (A.L.-B.); ivelasco@ua.es (I.V.); macien@umh.es (M.A.); 2 Biotechnology Department, Faculty of Sciences, University of Alicante, Carretera San Vicente del Raspeig s/n, 03690 San Vicente del Raspeig, Alicante, Spain; paula.saez@ua.es; 3 Gynecology Division, Faculty of Medicine, Miguel Hernández University, Carretera Alicante-Valencia s/n, 03550 San Juan de Alicante, Spain; 4 Department of Analytical Chemistry, Nutrition and Food Sciences, University of Alicante, Carretera San Vicente del Raspeig s/n, 03690 San Vicente del Raspeig, Alicante, Spain; jose.todoli@ua.es (J.-L.T.-T.); r.sanchez@ua.es (R.S.-R.)

**Keywords:** endocrine disrupting compounds, environmental exposure, heavy metals, human sperm, male fertility, occupational exposure

## Abstract

Heavy metals are endocrine disruptors which interfere with processes mediated by endogenous hormones of the organism, negatively affecting endocrine functions. Some studies have correlated heavy metal exposure with male infertility. However, the number of studies conducted on humans are limited. Therefore, the aim of this study is to summarize the current knowledge on how heavy metals influence human male fertility. Hence, three distinct databases were consulted—PubMed, Scopus and Web of Science—using single keywords and combinations of them. The total number of identified articles was 636. Nevertheless, by using the inclusion and exclusion criteria, 144 articles were finally included in this work. Results display that the development of adequate instruments for heavy metal assessment may play an important function in human male fertility diagnosis and treatment. Furthermore, clinical trials could be useful to confirm the role of heavy metals in human male fertility diagnosis. Overall, further research is required to fully understand the molecular and cellular basis of the influence of environmental and occupational exposure to heavy metals on human male infertility and reproductive outcomes.

## 1. Introduction

The definition of infertility was established by the American Society for Reproductive Medicine and it is defined as the failure to get pregnant after one year or more of regular sexual intercourse without the use of contraceptives. It can be due to an impairment of the capacity of reproduction, individually or with the partner. Globally, 15% of worldwide couples suffer infertility, which is equivalent to 48.5 million couples. Specifically, male partners are responsible for 20–30% of the overall infertility cases [1]. Further to that, those values change depending on the geographical region. The increased prevalence of infertility could be due to social factors, changes in seminal quality due to lifestyle habits (alcohol and tobacco consumption) and changes in sexual behavior [2]. The decline of male fertility is a worldwide matter of concern since available studies suggest a lower semen quality over the years. Worldwide data revealed a decrease in sperm concentration (−0.64 million/mL per year) from 1973 to 2011 [3]. Recent retrospective evidence and basic studies have shown relationships between the decrease in sperm quality and a poor diet, increased obesity rates and exposure to environmental toxins [4].

To address male infertility, a compilation of the patient’s sexual history, a complete physical examination, a serological test and a semen analysis are needed [5]. To evaluate male fertility and its management, the basic semen analysis has been considered fundamental. However, despite sperm analysis being routinely used, it cannot help to discriminate between infertile and fertile men. Thus, it is only useful to classify men as subfertile [6]. Due to the intra-individual variability, the World Health Organization [7] recommendation is the performance of two or three seminograms to obtain detailed information on the patient’s seminal parameters. Furthermore, the etiological factors affecting male infertility are varied, including environmental [8] and genetic factors, endocrine and immunological disorders, and obstructive lesions and infections in the male reproductive tract [9].

Conventional semen parameters are not useful, since 30–40% of the cases suffer unexplained or idiopathic male infertility [10]. Therefore, the routine sperm analysis assessment itself is not sufficient to test the fertilizing ability of spermatozoa. As a consequence, the development of new functional tests providing newer biomarkers of the sperm fertilizing capacity are needed [11]. Indeed, the physiology and functionality assessment of sperm should include studies on molecular biomarkers, such as the acrosome reaction, reactive oxygen species (ROS), DNA damage and chromatin structure [12]. DNA fragmentation plays an important role in subfertile patients [13]. This fact is a major matter of concern as DNA integrity is important for a proper embryo development [14] and it could be implicated in *in vitro* fertilization processes, as in the intracytoplasmic sperm injection, for instance [13]. The main culprits for DNA damage are an abortive apoptosis, problems during the protamine substitution in the spermatogenesis process and ROS production [13]. In this context, oxidative stress is considered an important cause of male infertility. Approximately 40% of patients show evidence of redox attack, thus exhibiting high levels of lipid peroxidation and oxidative DNA damage. In addition, it has been observed that a certain region in chromosome 15 may be especially vulnerable to oxidative attack and its genetic location is associated with male infertility [15].

Up-to-date literature links environmental contaminants and human reproductive health worries [16]. Male reproductive function is vulnerable to different environmental and occupational factors, of which only a few have been well identified. Different compounds are considered to be the main culprits for male fertility reduction, such as pesticides, dioxins, solvents and heavy metals. [17]. At this point, it is important to emphasize that environmental quality has recently decreased mainly due to anthropic activities that increase the level of environmental pollutants. Some of these pollutants can act as certain endogenous hormones and are therefore a cause for concern. They are the so-called endocrine disrupting compounds (EDCs) and are considered exogenous substances that are involved in the processes regulated by endogenous hormones of the organism, thus disrupting endocrine functions [18]. This group includes different substances, such as dioxins, bisphenol A and heavy metals. Although there is no authoritative definition for the term heavy metals [19], this group of elements has been considered as “naturally occurring metals having atomic number (Z) greater than 20 and an elemental density greater than 5 g cm^−3^” [20]. Therefore, a total of 51 different elements can be included in the category of “heavy metals”. In most cases, contaminated food is the main source of exposure to these species [21].

Since the human body has no biochemical pathways to detoxify them, heavy metal exposition leads to an accumulation in the body. For that, in the last century, the risks to health and development derived from the exposure to heavy metals have become a matter of interest. In particular, the aforementioned chemical species can affect male fertility by lowering the seminal quality, thereby causing infertility [22]. For example, copper (Cu) and chromium (Cr) were found in the semen samples of a father and his son from the “Land of Fires”, in Italy. This region is a highly environmentally polluted area, exposed to diverse chemicals and heavy metals [23]. Not only that, but the results obtained from this research showed alterations in the content of sperm nuclear basic proteins (SNBP) and a low DNA binding affinity. In addition, the son’s proteins showed unstable DNA binding, thus able to produce DNA damage [23]. Such evidence highlights the transgenerational inherited consequences of environmental-pollution exposition on molecular alterations in the sperm cell. Furthermore, men from highly contaminated regions showed higher zinc (Zn), Cr and Cu and lower iron (Fe) concentrations in semen, lower sperm motility and higher DNA damage than those that had not been exposed to environmental pollutants [24].

The main objective of this review is to perform a bibliometric and bibliographic analysis of the articles published during the last 25 years which studied the relationship between heavy metals and human male fertility. Additionally, this study aims to highlight the effects that heavy metals exert on the viability of the human sperm, identifying the elements involved, as well as the way they do it.

## 2. Materials and Methods

### 2.1. Search Strategy and Information Processing

First, generic searches were performed using the “Google scholar” portal (https://scholar.google.es/, accessed on 16 March 2021). This allowed us to identify important concepts relating human male fertility with heavy metal exposure, but also helped to select the final keywords to use them in a more comprehensive search through some specific scientific databases. The ultimate list of keywords derived from the “MeSH database” (Medical Subject Heading) and NLM (The National Library of Medicine), being the keywords “Male Fertility” and “Heavy Metals”. In addition, we added two more keywords to the initial ones, “Human Sperm” and “Human Spermatogenesis”.

A full search was performed on online databases related to the issue under study to achieve an accurate bibliometric and bibliographic analysis and to be aware of the bibliographic load indexed in each one of the online databases. The selected databases were Scopus, PubMed and Web of Science (WOS).

Finally, an advanced search was carried out in the WOS database, to identify the final documents of interest. The “Search all databases” option was selected during this research. The field tags “title” and “topic” were chosen and the information retrieval system “Boolean” model helped to identify the researches of interest for this review by using the keyword combinations mentioned above.

### 2.2. Selection of Relevant Studies and Data Analysis

The early search was performed, as described above, using the keywords “Male Fertility”, “Heavy Metals”, “Human Spermatogenesis” and “Human Sperm” in each one of the databases. An attached database was created in RefWorks to include the articles obtained from each search result.

**Figure 1:**
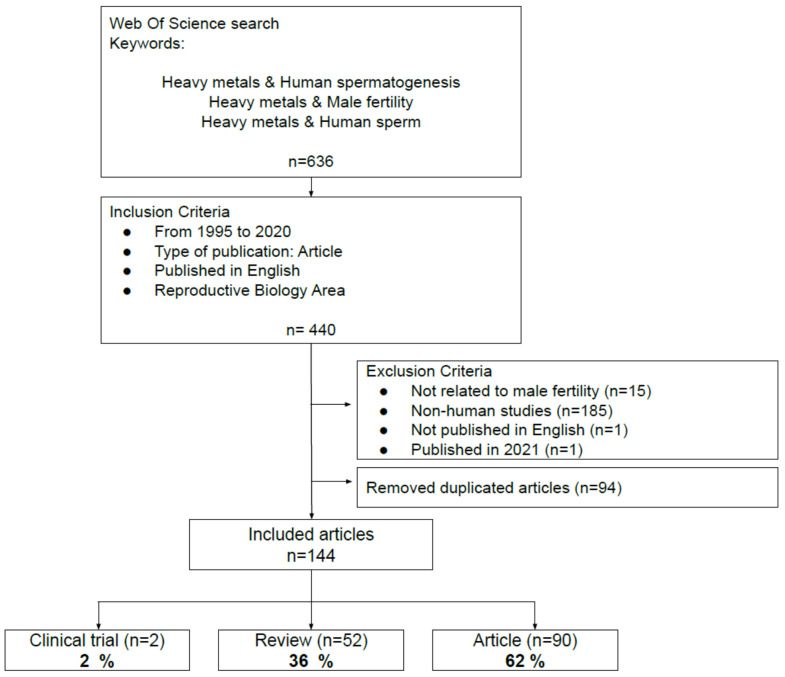
Flowchart summarizing the selection process of the articles included in the present review.

Afterward, another exploration strategy was proposed. It employed several keyword combinations in the different databases. The “Boolean” system allowed to identify the studies of interest for the present review, with “Heavy metals and Male Fertility”, “Heavy metals and Human Sperm” and “Heavy metals and Human Spermatogenesis” as keywords. In addition, we incorporated three inclusion criteria that helped to filter the results and served to select the documents: (i) a 25-year study period (1995–2020); (ii) articles from primary sources and indexed journals; (iii) studies published in English. WOS was the only database which allowed to select the research area (Reproductive Biology) as an inclusion criterion. This finding, along with the number of publications reported by each database, led to the selection of WOS for the bibliometric and bibliographic analysis to conduct this review. The following accorded exclusion criteria were applied: (i) studies not related to male fertility; (ii) non-human studies; (iii) studies not published in English; (iv) studies published in 2021. The elimination of duplicate articles (Mendeley) enabled us to build the final database. The final number of articles included in this review was 144. The flowchart in Figure 1 shows the WOS search and the steps followed to select the articles included in this review.

## 3. Results and Discussion

### 3.1. Compilation of Relevant Bibliographic Sources

Table 1 summarizes the total amount of articles from each keyword search. The advanced search also guaranteed that the resulting articles met all the selected inclusion criteria. Nonetheless, the search results displayed unrelated and non-specific articles for the matter of interest of this review. In spite of this, a low number of articles were found. As mentioned above, the WOS database was selected to perform the ultimate search because it allows to remove generic results.

Initially, a total of 636 articles were found after using the keywords in WOS, which became 581 after applying our inclusion criteria. Considering the reproductive biology area, 440 articles were included. According to the exclusion criteria, a total amount of 204 articles were discarded, which led to a total number of 236 publications.

Refworks and Mendeley enabled us to create a database. Then, 91 duplicate articles were excluded and, finally, a total of 144 articles were left—23% of the total articles initially found—forming the database to be ultimately analyzed. The analysis of these articles revealed that 52 were reviews (36%), 2 articles were clinical trials (2%) and 90 were articles (62%) (Figure 1). Supplemental Information is available with the information of every type of article reviewed (Tables S1–S3).

### 3.2. Bibliometric Analysis

**Figure 2:**
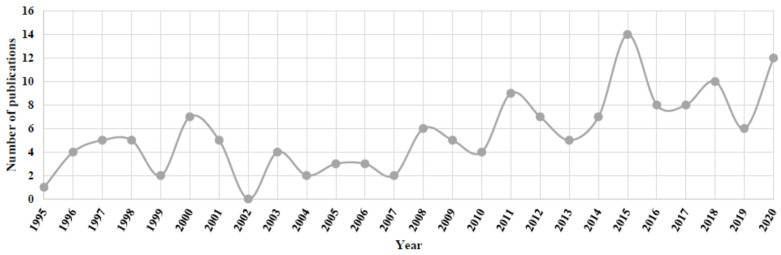
Number of publications as a function of the year (January 1995–December 2020) including all the articles from the database created after the application of inclusion and exclusion criteria.

The keyword search showed that the number of publications relating heavy metals, and human sperm and human spermatogenesis were lower than those related to male fertility. The analysis of the 144 articles selected in this review revealed that the highest value in the number of publications (*i.e.*, 14) was reached in 2015, whereas, in 2002 there were not publications within this field (Figure 2). Furthermore, an increase in the number of publications can be noticed after 2010. Specifically, 50% of the articles were published since 2012. These findings could be explained by an increase in male fertility problems related to heavy metals and/or because of the development of new sensitive multielement analytical tools.

The research on the relationship between heavy metals and human male fertility is a worldwide matter of interest, since the articles stemmed from diverse countries. However, when we focused on the authorship of the documents, the studies were conducted in countries with high environmental concerns, such as China and India.

### 3.3. Bibliographical Analysis

This section covers the analysis of all articles selected in this review, including (1) clinical trials, (2) reviews and (3) original articles. To facilitate reading, the chemical elements cited throughout this review and their corresponding chemical symbol are available in Table 2.

### 3.4. Analysis of the Clinical Trials

In terms of clinical trials, ref. [25] showed that a gene polymorphism in the δ-aminolevlinic acid dehydratase (ALAD) would be responsible for individual susceptibility to Pb poisoning. The results suggest that the ALAD-2 polymorphism could protect semen parameters such as total sperm count and sperm concentration in workers whose blood Pb levels are higher than 40 µg/dL. Additionally, to determine which trace elements were attached to the spermatic DNA, an analytical method was developed [26]. The results show the following affinity order: Aluminum (Al) > Lead (Pb) > Cadmium (Cd). In oligozoospermic and teratozoospermic patients, the concentration of Al attached to DNA was significantly higher than in normozoospermic.

### 3.5. Analysis of the Reviews

#### 3.5.1. Heavy Metals Effects in the Reproductive Processes

Heavy metal exposure has been identified as an influential factor on male sperm production and fertility [27,28,29]. However, the mechanisms that alter the reproductive processes are complex. The toxicant effects can be directly produced by the action on the reproductive organs, or, indirectly, by impairing the hormonal regulation [30]. Moreover, different biological matrices are used to evaluate male reproductive risks. The biological matrices usually analyzed are blood, serum, semen, seminal plasma, urine, or hair. Heavy metals are found at higher concentrations in blood or urine [17].

There is some controversy about the impact of heavy metal exposure on biological matrices. Thus, semen had been considered a less informative marker of occupational exposure. Furthermore, it is recognized that the heavy metals determination in spermatozoa cells is not a more enlightening marker of the occupational (or non-occupational) exposure than the biological and traditional monitoring by urine and blood. In contrast, for some specific research and clinical purposes, the study of spermatic cells and semen could be appropriate [17]. Following this idea, a recent study from the EcoFoodFertility initiative concluded that semen could be considered an early biomarker of environmental exposure to Zn, Cr and Cu, since higher concentrations of these elements had been demonstrated in men residing in areas with high environmental impact [24]. In particular, the sperm DNA fragmentation assessment has been recently proposed as a marker of air pollution [31]. Regarding environmental pollution, a retrospective observational study in China carried out during the COVID-19 outbreak showed higher susceptibility to poorer sperm motility [32]. Due to that, air pollution and COVID-19 may be currently considered hazardous to male fertility. Interestingly, according to a recent study performed in China, a higher percentage of apoptotic cells in the testis was present in COVID-19-infected patients and a decreased sperm concentration (39.1% of COVID-19 patients) was found in semen [33]. Those findings highlight that, in air polluted countries such as China, COVID-19 may exacerbate the effects of environmental contaminants, leading to impaired male fertility.

Most EDCs have intrinsic estrogenic or androgenic activity, being the gonads the targets of most of these compounds [34]. Different endpoints are used to study the masculine reproductive function, and the seminal quality and the endocrine and cellular secretion function have been the most frequently used [35]. Among others, Cd, arsenic (As) and Pb were recently identified as major toxicants affecting the reproduction function. The toxicity mechanisms include oxidative stress, inflammation, apoptosis and endocrine disruption [36]. Cd effects have been widely studied. Different toxicity mechanisms are linked to Cd exposition, including inflammation, cytotoxicity, oxidative stress, apoptosis and disruption of signaling pathways, which regulate the reproductive functions [37].

Almost all the masculine reproductive tract units are targets of EDCs. Testes are the direct target of a lot of toxicants, such as Cd [38]. Elements such as Cd, mercury (Hg) and Pb produce a dysfunction in Sertoli cells (SCs) [35]. Toxicants affecting Leydig cells can cause anomalies in the testosterone secretion, which results in an impaired SCs function and in a defective spermatogenesis. Moreover, the Leydig cells and SCs affectation can lead to the seminiferous epithelium reduction [39]. In addition, the affectation of the spermatogenesis process can occur through the alteration of the adhesion between germ cells or SCs [35]. Most of the studies where the role of the SCs is assessed have been carried out *in vitro*. Note that comparisons between the results found *in vitro* and *in vivo* studies are difficult [40]. Besides, a brief exposure of the testes to Cd could alter the molecular defense system. Cd reaches its molecular targets (FAK and cadherins), impairing the cell adhesion function and interrupting the spermatogenesis process [34].

There is evidence that certain toxicants interact with the secretion of hypothalamic releasing factors, luteinizing hormone (LH) and follicle-stimulating hormone (FSH), all of which play a major role in sperm quality [30]. Besides, the endocrine-hormone levels could inform about the functionality of the testicular cells, although it is not a well considered biomarker [35]. The hypothalamic–pituitary–testis (HPT) axis is also an EDC target [37,41], which results in the alteration of the physiological function of the testis [42]. During the steroidogenesis process, heavy metals disrupt the androgen production. This could be mediated by receptors or by direct effects on gene transcription.

Furthermore, EDCs induce oxidative stress, which plays an important role in male infertility [22]. In physiological conditions, ROS are molecules that affect normal sperm functionality, whereas seminal plasma contains antioxidant molecules and biomolecules that help to maintain the balance [43]. However, exposure to heavy metals increases the production of ROS and decreases the antioxidant defenses. This fact induces alterations in SCs, such as DNA damage, lipid peroxidation and, ultimately, apoptosis [35]. Patients with an elevated ROS production in semen may benefit from an antioxidant therapy. Antioxidant supplements can be separated in two categories, synthetic or natural. Synthetic antioxidants are chemically synthesized and isolated compounds, while natural antioxidants are spontaneously present in foods. The consumption of natural antioxidants seems to increase the total antioxidant capacity [44]. In agreement with this, a diet rich in carotenoids, for instance, could improve the sperm motility and morphology [45]. In addition, lower rates of aneuploid sperm were correlated with dietary folate intake [46]. Hence, dietary supplementation with antioxidants may be interesting to ameliorate seminal quality, thus improving male reproductive health.

#### 3.5.2. Sources of Heavy Metals Exposure

Localized exposure to heavy metals, e.g., due to one’s occupation, or to one’s lifestyle habits, is the most common type of exposure. Generally, the human population is exposed to heavy metals voluntarily. The voluntary way of exposure to these toxicants can be by oral supplementation. On the contrary, involuntary exposure can be through the intake of contaminated water or food. Nowadays, the environment is a matter of concern because heavy metals are widely distributed [47].

##### Food Exposure

Although diet, which includes daily intakes of antioxidants, may be useful to improve the ROS production in semen, it is also a common way to consume heavy metals.

The intake of contaminated food is a usual source of exposure to heavy metals, as described above. Contamination can happen during the handling of food and its processing. Contaminated soils may also contribute to the pollution of food of both vegetable and animal origin [22]. Drinking water contaminated with As has arisen as an important health problem in Asia, India and China, among other countries [48]. Focusing on food, fruits may contain Cu, Pb and Zn at high concentrations [49]. Moreover, strawberries, dates, spinach and cucumber contain Cd, in addition to the above-mentioned elements [50]. In milk, meat and meat-derived products, elements such as As, Cd and Pb were detected [51]. In seafood, inorganic As was found in algae [52]. Additionally, in cereals such as Indian rice, As, Pb, Cd and Hg were found, although the values did not exceed the maximum residue limits [53].

##### Environmental Exposure

Environmental exposure includes the exposure to environmental pollutants, including tobacco smoke. They have the potential to alter the male reproductive system, thus worsening the capacity of conceiving a healthful offspring [54]. The environmental pollutants also have transgenerational genetic effects, which may affect future generations. This problem is currently an important matter of concern [28]. Studies with environmentally realistic exposure levels to heavy metals and their impact on male reproductive outcomes are scarce, while those with high occupational-exposure levels had been widely documented. However, low-exposure effects have been evidenced, being stronger for elements such as Cd, Pb and Hg [55].

A meta-analysis found that the environment pollution reduced sperm motility. There is some concern about the decline of male fertility in metropolitan areas [56] and, especially, in countries with a higher risk of exposure, such as Nigeria [57]. The sperm concentration in the African population has decreased significantly by 73% during the last 50 years [58]. This problem is caused by multiple exposures to environmental toxins, including Cd and Pb. Future studies should analyze the relationship among the nature of the duration, the intensity of the exposure and the degree of infertility, as the effect on male fertility depends on those parameters [56]. Although the influencing factors are multiple, exposure to pesticides and heavy metals is the main culprit [58].

Heavy metals such as Pb or As are present in tobacco smoke, as well as Cu, Cd [59], Cr and nickel (Ni) [60,61]. As mentioned above, some studies suggest a relationship between tobacco smoke and adverse reproductive outcomes [54]. Hg is another highly toxic environmental pollutant. Recent research found that it may cause impairments in the reproductive function, leading to deformations in the seminiferous tubules and in Leydig cells and giving rise to a final testicular deterioration [62].

Environmental toxicants lead to ROS formation. This stress is associated with several deleterious effects, such as testicular apoptosis, erroneous protamination, abnormal sperm functionality and viability, and oxidative DNA damage, causing male infertility [15,63]. This pro-oxidative microenvironment is produced, among others, by certain heavy metals (Cd, Pb, Cr, manganese (Mn), Hg, Zn and Cu). Cd is a widely studied heavy metal, which increases the activity of ROSs and induces changes in the enzymatic activity systems and inflammatory reactions [64]. As mentioned above, Cr and Cu are two heavy metals responsible for an altered content of SNBP in men environmentally exposed to pollutants. In addition, they are correlated with spermatic DNA damage due to an unstable DNA binding [23]. Further to this, an altered protamines/histones ratio was found in 84% of men residing in air polluted areas and, thus, exposed to these elements. Moreover, a different DNA binding pattern was found in men with a normal protamines/histone ratio. Unexpectedly, in those samples, SNBP were involved in the oxidative damage present in the spermatic DNA [65]. Some studies performed on animal models, such as *Mytilus galloprovincialis* L., yielded similar conclusions as those drawn for humans. For instance, in the presence of Cd, protamine-like proteins (PL) as PL-II suffered complete conformational changes, promoting a different DNA-binding mode. This resulted in affecting the sperm chromatin organization, which is crucial for a successful fertilization [66].

Nevertheless, further studies focusing on the molecular mechanisms are needed. In addition, the epigenetic modifications implicated in smoking cigarettes needs to be known, since tobacco smoke is a widely present environmental contaminant. More importantly, as these modifications are heritable, it would be interesting to study the transgenerational effects of tobacco smoke through the paternal line [67,68].

##### Occupational Exposure

Hazardous occupations in reproductive terms are evaluated based on years of service and agents of exposure. Normally, exposure data and other cause–effect parameters are insufficient to indicate which chemical factor is responsible for reproductive dysfunctions [30]. Workers exposed to heavy metals present a high risk of suffering a reproductive dysfunction [69]. Pb was one of the most studied elements which had a negative relationship with male fertility. Others, such as Cd and Cr, also have negative effects on fertility [70]. It must be borne in mind that the effects overlap due to multiple exposures. Observed adverse effects include reduced fertility, poor seminal quality, enhanced risk of lower birth weight, miscarriages and permanent sterility [69].

Some reports have suggested a decline in sperm concentration and an increase in reproductive disorders over the past 50 years. Occupational exposure to heavy metals such as inorganic Pb and Hg occurs in professions related to metal smelting or welding, or boron mining [71]. Specifically, welding is considered as one of the main occupational agents that negatively affects male fertility [72]. Welding is an occupation that endangers workers, because of the exposure to fumes and dust that contain, among others, metals. In this area, the exposure level in the western world has declined, but some specific workers still work in such industries, so fertility remains threatened [72]. It can be concluded that occupational exposure to metals is associated with an impaired male reproductive function. The evidence of this effect is stronger for some metals, such as Pb, Mn and Hg, than for others, such as Cd, Cr and Ni [10].

### 3.6. Analysis of the Articles

The inspection of the articles which studied the relationship between heavy metals and seminal parameters led us to generate four categories: (Section 3.6.1) *in vitro* studies where sperm was exposed to heavy metals, (Section 3.6.2) articles in which the study subjects were men from the general population, (Section 3.6.3) articles where the study subjects were occupationally or environmentally exposed men and (Section 3.6.4) articles with men attending centers for assisted reproduction as study subjects.

#### 3.6.1. *In Vitro* Studies Exposing Sperm to Heavy Metals

A low number of articles (*n* = 8) studied the *in vitro* effects of specific heavy metals on sperm parameters. Generally, all the works showed that heavy metals generate a negative impact on sperm functions when they were incubated *in vitro* under specific concentrations.

It was shown that the mannose receptor expression in capacitated sperm was inversely correlated with Pb levels [73]. Due to that, this receptor could be a biomarker reflecting the effects of heavy metals on sperm fertility capability. Other articles studied the effect of specific metal ions on sperm functionality. Metals such as Hg, Pb, tin (Sn), silver (Ag), bismuth (Bi) and indium (In), showed an inhibition of sperm creatine kinase, probably causing male infertility [74]. Some motility parameters were significantly lowered after the exposure to high concentrations of copper sulfate (CuSO_4_) and cadmium chloride (CdCl_2_) [75]. In addition, lead was found to inhibit human sperm functions by diminishing the levels of the sperm intracellular cyclic adenosine monophosphate (cAMP), calcium and protein tyrosine phosphorylation [76]. In addition to that, Fe, at a concentration of 5 parts per million (ppm), could induce lipid peroxidation, leading to an inhibition of sperm motility [77]. Lastly, a decrease in progressive and hyperactivated sperm motility was detected after the *in vitro* incubation of spermatozoa with 10 µM CdCl_2_ for 24 h [78]. In addition, it also affected the physiological spermatic response to progesterone and induced the spontaneous acrosome reaction in human sperm.

#### 3.6.2. Men from General Population as Study Subjects

Only a small number of scientific articles had men from the general population as study subjects (*n* = 4) (Table 3). In general, the most used biological matrix was serum. Heavy metals (Mn and Pb) were observed to negatively affect the normal morphology of sperm [79,80]. In addition, the Cu/Zn ratio was found to be higher in the group with abnormal progressive motility [81]. Meanwhile, it was shown that Pb and Cd were present in the semen of men from the general population. The high Pb content observed in some semen samples may be due to a high traffic density [82].

#### 3.6.3. Occupationally and Environmentally Exposed Men as Study Subjects

A good number of articles (*n* = 21) had occupationally and environmentally exposed men to heavy metals as study subjects (Table 4). In this case, a wide variety of biological matrices were analyzed to assess the heavy metal concentration, such as hair, semen, seminal plasma, serum and urine. Generally, in the occupational studies, the groups were divided in occupationally exposed men and non-occupationally exposed men (control). When the environmental contaminants were assessed, the experimental groups showed greater heterogeneity. We found articles which studied infertile men [83,84,85,86], comparing or not comparing them with a control group and environmentally exposed men from the general population as study subjects [87,88]. It should be noted that, after analyzing all the scientific articles and as specified in the following paragraphs, the most studied heavy metals were Pb, Hg and Cd.

In terms of Pb, men exposed to this heavy metal showed greater concentrations in blood and semen, which were correlated with lower sperm motility [72]. Another study displayed that the spermatic quality was lower in the exposed group than in the control, showing differences in the spermatic concentration, motility, viability and abnormal morphology [90]. In addition, other authors found lower sperm vitality, sperm membrane surface alterations, lower sperm velocity, gross and forward progressive motility in the exposed group [91]. Similarly, lower sperm count, density, motility and semen volume along with an increase in the incidence of sperm abnormalities and prolonged liquefaction time, were observed in the Pb exposed group [92]. Meanwhile, increased risk of teratozoospermia was also associated with high blood and semen Pb levels [91].

Focusing on Hg, a high concentration of this chemical agent was found in semen samples of men working at a thermometer manufacturing plant [93]. Furthermore, Hg also inhibited the spermatogenesis process in some Hong Kong males [83]. In their study, it was highlighted that a daily Hg intake of 0.3–0.7 milligrams per kilogram of body weight could be sufficient to inhibit spermatogenesis. Furthermore, a significant correlation was found between male subfertility and the level of this metal in male hair, showing a 40% increase in subfertile males, compared to fertile males of similar age [84].

Cd exposure showed controversial results and no significant correlation between seminal Cd concentrations and traditional semen parameters was found. In addition, there was no statistical relationship between the fecundity status of the patients and Cd concentration. For normozoospermic patients, the mean Cd concentration in seminal plasma was higher in the group of smokers than in the group of non-smokers [94]. In contrast, for men with no occupational exposure to Cd, a high concentration of this heavy metal was positively correlated with a reduction of the semen progressive motility, volume and morphology [87]. It is worth mentioning that increased Cd and Pb concentrations have been found in the semen of occupationally exposed subjects [95]. Those concentrations were positively associated with an increased number of defects in sperm and with the levels of some seminal oxidative stress markers. Conversely, an inverse correlation of Cd and Pb concentration in semen has been identified with sperm motility [85]. Besides these findings, environmental and occupational exposures to both toxicants were associated with reduced sperm normal forms, motility, viability, sperm count and detectable levels of Pb and Cd in seminal plasma [96].

#### 3.6.4. Men from Assisted-Reproduction Centers as Study Subjects

Articles including men assisting in reproduction clinics or centers as study subjects were also examined for the present review (*n* = 13) (Table 5). Within this category, the heavy metal content was measured in different biological matrices (whole blood, blood plasma, serum, seminal plasma, semen, urine, etc.), with seminal plasma and blood being the most studied ones.

It has been observed that Cu levels in blood plasma were higher in males with normal sperm morphology and function than in azoospermic, asthenoteratozoospermic and severely oligoasthenoteratozoospemic men. In addition, Cu levels in seminal plasma were positively correlated with sperm count and morphology, while they were negatively correlated with semen volume. Conversely, levels higher than 1.5 μg/L of Cd in whole blood were associated with a significant decrease in sperm count [104].

It was noticed that increased blood levels of Pb and FSH were associated with low semen quality [105]. High levels of Pb and Cd were observed in infertile men. In addition, a significant negative correlation was found between Cd and Pb levels in seminal plasma and the sperm motility and concentration in oligoasthenospermic men [106]. In addition, low-quality semen participants had significantly higher Cd and barium (Ba) concentrations in semen [107].

In a meta-analysis, where an infertility and a control group were studied, they found that high Cd semen content was a causative factor of infertility [117]. This heavy metal was widely spread in infertile patients, leading to low sperm quality. Thus, the Cd level in semen could be a good indicator of sperm quality. Moreover, other elements have been widely analyzed. Selenium (Se) serum levels showed a higher concentration in the fertile group, while higher levels of Pb and Cd in seminal plasma and serum were observed in the infertile group [108].

The relationship between heavy metals and the reproductive outcomes of patients visiting centers for assisted reproduction was also assessed. However, the number of articles studying this topic was limited (*n* = 4). Table 6 shows the articles included in this review. The paternal pre-conception exposure to high levels of Ba, tungsten (W) and uranium (U) in urine lead to a shorter gestational age at delivery [118]. The male’s blood Pb concentration was correlated with reduced fecundability odd ratios [119]. Besides, the seminal As concentration was significantly correlated with a better quality of cleavage embryos. A higher Se concentration in the seminal plasma was related to greater pregnancy and live-birth probabilities [120]. Finally, a high content of Zn in seminal plasma could have a positive role in embryo transfer and in the IVF outcomes [121].

## 4. Conclusions

**Figure 3:**
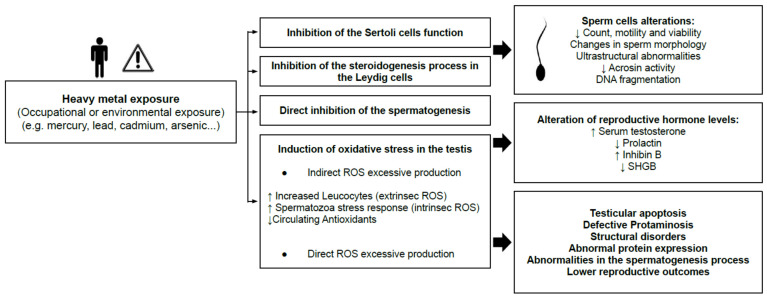
Summary of the effects of heavy metal exposure on men’s reproductive health. ROS, reactive oxygen species; SHBG, Sex Hormone Binding Globulin.

The masculine reproductive function is vulnerable to several environmental and occupational hazards. Those compounds have not been totally identified, but substances as dioxins, polychlorinated biphenyls, phthalates, polycyclic aromatic hydrocarbons, pesticides, alkylphenols, bisphenol A and heavy metals (Hg, Cd, Pb, As, Pb, etc.) are some of them [21]. Exposure to heavy metals, which can happen through occupational or environmental exposure, is, nowadays, a threat to reproductive health (Figure 3).

Those chemicals can mimic hormonal functions, so they are also known as endocrine disrupting compounds (EDCs). Man exposed to heavy metals have lower seminal quality, which is related to infertility or to a subfertility status. The negative effects are produced through the inhibition of the normal function of Sertoli and Leydig cells, the disruption of spermatogenesis and by the induction of oxidative stress in testes (indirect and direct reactive oxygen species production). Abnormal parameters of sperm count, motility, viability, morphology and DNA fragmentation, together with alterations in hormones levels, can also be found in men exposed to EDCs. Along with the design of appropriate therapeutic strategies to prevent located exposures to heavy metals, defects and deficiencies need to be considered. This is very relevant to overcome male factor fertility problems. To combat this, it is necessary to develop adequate tools (sensitive, non-invasive, fast and reliable) for heavy metal assessment in order to clear the diagnosis and improve seminal parameters.

Human studies are scarce and there is a lack of homogeneity in the methodology. In addition, the nature of the biological matrix (urine, blood, blood plasma, seminal plasma, semen, etc.) shows different results, making it difficult to choose one as the best option due to the different information that they provide. However, it should be noticed that the sperm content could be an accurate indicator of the impact of certain toxic substances on the reproduction potential.

Nevertheless, despite the discrepancies among the different methodologies observed in the studies, they generally emphasize the existence of a significant correlation between exposure to heavy metals and lower seminal quality, related to a lower fertility rate. Further to this, we found evidence suggesting that scientific research has proved that Pb and Cd are the analytes that most negatively affect seminal quality. Both have been correlated by different researchers with lower sperm concentrations, an abnormal spermatic morphology and lower sperm viability.

Further investigation is needed to completely understand on a molecular and cellular basis, how environmental and occupational exposure to heavy metals is related to infertility and, ultimately, its impact on reproductive outcomes. Due to the available facts, a greater collaboration between clinicians, epidemiologists and scientists is needed to identify the environmental chemicals, as well as the molecular and cellular processes, responsible for reproductive problems. If the aforementioned information is verified, it would be highly useful to introduce a heavy metal assessment in the biological analysis of samples of patients attending an assisted-reproduction center, allowing a personal diagnosis and prognosis and preventing men infertility. This would improve the success of the assisted reproductive techniques.

## Figures and Tables

**Table 1:** Number of publications in each database according to the different keywords utilized after applying the inclusion criteria.

Keyword	Pubmed	Scopus	WOS
Male Fertility	38,256	186,504	52,261
Heavy metals	345,873	513,286	199,667
Human Spermatogenesis	9211	54,300	199,667
Human Sperm	35,460	133,717	46,870
Heavy metals and Human Spermatogenesis	101	1144	69
Heavy metals and Human Sperm	573	2972	222
Heavy metals and Male Fertility	428	2722	290

**Table 2:** Chemical symbols of the elements and cited in this review.

Symbol	Chemical Element	Symbol	Chemical Element
Ag	Silver	Mo	Molybdenum
Al	Aluminum	Ni	Nickel
As	Arsenic	Pb	Lead
Ba	Barium	Pt	Platinum
Be	Beryllium	Sb	Antimony
Bi	Bismuth	Sc	Scandium
Ca	Calcium	Se	Selenium
Cd	Cadmium	Sn	Tin
Co	Cobalt	Sr	Strontium
Cr	Chromium	Te	Tellurium
Cs	Cesium	Ti	Titanium
Cu	Copper	Tl	Thallium
Fe	Iron	U	Uranium
Hg	Mercury	V	Vanadium
In	Indium	W	Tungsten
Mg	Magnesium	Zn	Zinc
Mn	Manganese		

**Table 3:** Articles studying the relationship between heavy metals and reproductive parameters in study subjects from general population.

Ref.	Study Groups	Biological Matrix	Detection Method	Metal Profile	Main Findings
[79]	Men from six cities of China (*n* = 1179)	SE	AAS	Zn and Cu	- The progressive motility parameters showed differences between groups. - Higher risk of asthenozoospermia with lower serum Zn levels. - Higher Cu/Zn ratio in the group of men with abnormal progressive motility.
[80]	Croatian men with no occupational exposure (*n* = 240)	SP, B and SE	BPb and BCd by AAS. SECu and SEZn by F-AAS. Se by ETA-AAS. SEPZn by bichromatic analyzer.	BCd and BPb, SECu, SEZn and SESe. Zn on SP	- Associations of BPb with reproductive parameters indicated a lead-related increase in immature sperm concentration and in abnormal sperm morphology.
[81]	Healthy volunteers from China (*n* = 1179)	SE	ICP-MS	Mn	- Increased teratospermia risk with serum Mn concentrations higher than 19.40 μg/L.
[82]	Samples (*n* = 50) from the general population of Lucknow (India)	S	GFAAS	Cd and Pb	- Pb (67.54 μg/L) and Cd (0.96 μg/L) were present.

AAS, atomic absorption spectroscopy; ETA-AAS, electrothermal-atomic absorption spectrometry; F-AAS, flame-atomic absorption spectroscopy; GFAAS, graphite furnace atomic absorption spectroscopy; ICP-MS, inductively coupled plasma-mass spectrometry. B, blood; S, semen; SE, serum; SP, seminal plasma; Cd, Cadmium; Cu, Copper; Mn, Manganese; Pb, Lead; Se, Selenium; Zn, Zinc.

**Table 4:** Articles studying the relationship between heavy metals and reproductive parameters in occupationally or environmentally exposed study subjects.

Ref.	Exposure	Study Groups	Biological Matrix	Detection Method	Metal Profile	Main Findings
[83]	E	Infertile men (*n* = 117) and fertile men as control group (*n* = 67)	H	ICP-MS	Hg	- A daily Hg intake of 0.3–0.7 mg per kg of body weight may be sufficient to inhibit the spermatogenesis process. - There was a significant correlation between male subfertility and the level of Hg in the hair of males between the ages of 25 and 75.
[84]	E	Infertile men (*n* = 117) and fertile men as control group (*n* = 49)	H	ICP-MS	Mn, Fe, Zn, Cu, Cd, Pb, Ni, Hg and Cr	- Compared to fertile males, subfertile males had up to a 40% of Hg in hair.
[85]	E	Infertile men (*n* = 150) and fertile men as control group (*n* = 60)	S	AAS	Pb and Cd	- Inverse correlation of both toxicants with sperm motility.
[86]	E	Men recruited from a reproductive medicine center (*n* = 746)	SP	ICP-MS	Al, Cr, Mn, Fe, Co, Ni, Cu, Zn, As, Se, Mo, Cd, Sn, Sb, W, Tl, Pb and U	- SPAs was negatively associated with total and progressive sperm motility. - Inverse correlations between Cd quartiles and progressive and total sperm motility. - Positive correlations between Zn quartiles and sperm concentration, between Cu and As quartiles and the percentage of tail DNA, between As and Se quartiles and tail extent and tail distributed moment, and between Sn and the percentage of necrotic spermatozoa.
[87]	E	Men from the general population (*n* = 587)	SE	ICP-MS	Cd	- A higher Cd concentration may reduce semen volume, progressive motility and morphology among men without occupational exposure to Cd.
[88]	E	Normozoospermic men from general population (*n* = 62)	SP and U	F-AAS, ETA-AAS and HG-AAS	Zn, Cu, Cd, As, Se and Pb	- Urinary Cd concentrations were negatively associated with sperm viability. - The normozoospermic group had significantly lower seminal plasma Cu concentrations than the abnormal group.
[89]	O	Exposed men (*n* = 5) and fertile and unexposed men as control group (*n* = 8)	B and S	AAS	Pb	- In the exposed group, the Pb concentration in blood and semen were significantly higher. - BPb and SPb concentrations were negatively associated with motility.
[90]	E	Exposed group (*n* = 20) and non-exposed group (*n* = 27) as control group	B and S	GFAAS	Pb	- The PbB concentration was significantly greater in the exposed group, as well as the PbS. - In the exposed group, there was a significant correlation between PbS and PbB. - Overall, the sperm quality was lower in the exposed group than in the non-exposed group. There were significant differences in motility, concentration, viability and abnormal morphology.
[91]	O	Low occupationally exposed group (*n* = 30) with 7–10 years exposure for 8 hours per day and high exposed group (*n* = 50), with more than 10–15 years of lead exposure.; non-occupationally exposed as control group (*n* = 50)	B and S	AAS	Pb	- After exposure, there was evidence of diminution in sperm cell production. - Low sperm vitality and hypoosmotic swelling percentage with high malondialdehyde content and altered seminal plasma ascorbate level indicated damage of sperm cell surface. - Altered sperm membrane surface. - Lower sperm velocity, gross and forward progressive motility. - High BPb and SPb levels were associated with teratozoospermia.
[92]	O	Low exposed group with 7–10 years exposure (*n* = 30) and high exposed group with exposure period of more than 10–15 years (*n* = 50); 40 non-occupationally exposed as control group	B and S	GFAAS	Pb	- Exposure lead to a lower sperm count and density, motility and volume along with an increased incidence of spermatic abnormalities and prolonged liquefaction time. - Also, exposure showed structural alteration of sperm cells, significantly low sperm viability, high sperm membrane lipid peroxidation rate, altered dehydroascorbate levels and low hypoosmotic swelling test (HOST) percentage. - Both BPb and SPb were significantly higher among the factory workers.
[93]	O	Husbands of women going though infertility treatment (*n* = 80) and workers from a thermometer manufacturing plant as exposed control group (*n* = 7)	S and U	CV-AAS	Hg	- The urinary concentration of Hg in exposed subjects was similar to non-exposed subjects. - Workers occupationally exposed to Hg showed elevated levels in semen.
[94]	O	Men with proven fertility (*n* = 12), normozoospermic patients (*n* = 44), unselected patients of an infertility clinic (*n* = 118) and industrial workers with occupational exposure to cadmium (*n* = 2)	SP	ETA-ASS	Cd	- No significant correlation was found between SPCd levels and traditional semen parameters. - Mean Cd concentration in seminal plasma of normozoospermic patients was higher in the group of smokers.
[95]	O	Male tea garden workers (*n* = 200) and samples from age-matched donors as control group (*n* = 200)	S	F-AAS	Pb and Cd	- In the high-concentration groups, Pb and Cd concentrations showed positive associations with a higher number of defects in sperm and with the level of markers of seminal oxidative stress.
[96]	E and O	Male partners (*n* = 300) of couples investigated for infertility	SP	GFAAS	Pb and Cd	- In SP, the levels of PB and Cd were associated with reduced sperm viability, count, motility and normal forms.
[97]	E	Infertile men with intramedullary nailing prosthesis (IMN) (*n* = 60) and age-matched healthy men as control group (*n* = 30)	SP	ICP-MS	Co, Cr and Mo	- Individuals with IMN had high concentrations of Co, Cr and Mo in SP. This led to a greater spermatozoal apoptotic activity.
[98]	E	Male partners in couples from a reproductive medicine center (*n* = 1247)	U	ICP-MS	As, Cd and Pb	- Urinary As, Cd and Pb were associated with oxidative stress markers (8-OHdG, HNE-MA and 8-isoPGF2α), which were also associated with impaired semen quality.
[99]	E and O	Infertile men (*n* = 74) and fertile men as control group (*n* = 76)	B, SP and U	ICP-MS	As, Mn, Co, Cd, Pb, Zn and Se	- Heavy metals levels in blood were not related to a lower fertility in Lebanese men.
[100]	E	Exposed men (*n* = 30) and control groups: men from towns 100 km away (*n* = 32, control group one) and 200 km away (*n* = 33, control group two)	SP	GFAAS	Cd, Cr and Cu	- SP Cr values displayed a significant negative correlation with normal morphology sperm rate, motility and rapid progressive sperm motility. - SP Cu values also displayed a negative correlation with normal morphology sperm rate.
[101]	E	Non-smoking males visiting infertility clinics (*n* = 333)	U	ICP-MS	Cd, Cr, Mn, Fe, Co, Ni, Cu, Zn, Sr, Mo, Sn, Sb, Ba, W, Tl, Pb and U	- Cd modified the association between polycyclic aromatic hydrocarbons (PAH) and pyrene with seminal quality parameters.
[102]	O	Workers from plants with a range of exposure to Pb (from no exposure to moderate Pb exposure) (*n* = 98) and no likely exposure (*n* = 51)	SP, B and SE	AAS	BCd, SZn, SfZn, Scu	- BPb was significatively associated with a decrease in sperm density, viability and progressive motility. It was also associated with an increase in abnormal sperm head morphology, an impaired prostate secretory function and a decreased serum testosterone and estradiol levels. - BCd was associated with a decrease in sperm motility. It also was associated with an increase in abnormal sperm morphology and with increased levels of serum testosterone. - Moderate exposures to Pb and Cd could significantly diminish human semen quality without impairing the reproductive endocrine function.
[103]	O	Welders workers (*n* = 57) and 57 controls (*n* = 57)	B	ICP-MS	Ni and Cr	- Ni was associated with a higher percentage of sperm with tail defects. - Cr was negatively correlated with the spermatic concentration.

E, environmental; O, occupational; B, blood; H, hair; S, semen; SE, serum; SP, seminal plasma; U, urine; AAS, atomic absorption spectroscopy; CV-AAS, cold vapor-atomic absorption spectroscopy; ETA-AAS, electrothermal-atomic absorption spectroscopy; F-AAS, flame-atomic absorption spectroscopy; GFAAS, graphite furnace atomic absorption spectroscopy; HG-AAS, hydride generation-atomic absorption spectroscopy; ICP-MS, inductively coupled plasma-mass spectrometry; Al, Aluminum; As, Arsenic; Ba, Barium; Cd, Cadmium; Cr, Chromium; Cu, Copper; Co, Cobalt; Fe, Iron; Hg, Mercury; Mn, Manganese; Mo, Molybdenum; Ni, Nickel; Sb, Antimony; Se, Selenium; Sn, Tin; Sr, Strontium; Tl, Thallium; U, Uranium; W, Tungsten; Zn, Zinc.

**Table 5:** Articles studying the relationship between heavy metals and reproductive parameters in study subjects from ART.

Ref.	Study Groups	Biological Matrix	Detection Method	Metal Profile	Main Findings
[104]	Men with a low seminal quality (*n* = 42) and normozoospermic cases (*n* = 10)	WB, SP and BP	Polarized Zeeman AAS	Zn, Cd, Pb, Cu	- Subjects with lower levels of SP Zinc had significantly lower numbers of normal sperms. - Positive correlation between Zn level in BP and total motility, rapid progressive motility and morphology; a negative correlation was found with seminal pH. - Cu levels in BP significantly higher in males with normal sperm morphology and function than AZS, asthenoteratozoospermia (ATZS), and severe oligoasthenoteratozoospemia (SOATZS). - A positive correlation was found between SP Cu levels and sperm count and morphology; a negative correlation was detected with semen volume. Higher levels of Cd in WB than SP were associated with a significant decrease in sperm count, especially at a concentration greater than 1.5 μg/L. - A negative correlation between Cd levels in WB and rapid progressive motility and morphology, in SP and rapid progressive motility.
[105]	Low-quality semen group (*n* = 59) and high-quality semen group (*n* = 95) from a reproductive medical center.	B	ICP-MS	Pb, Cd, Cr, Se, Fe, Cu and Zn	- Higher BPb levels were correlated with lower semen quality. - Increased BPb levels (higher than 50 μg/L) had an 11-fold probability of having poor semen quality. - There was no relationship between BPb and FSH levels.
[106]	Infertile male partners (oligospermia *n* = 17, oligoasthenospermia *n* = 12, asthenospermia *n* = 12, azoospermia *n* = 9) and 50 men with proven fertility as a control group	SP	AAS	Cd and Pb	- Infertile men had increased levels of Pb and Cd. - Sperm from oligoasthenospermic men showed lower motility and concentration. This was significantly correlated with SP Cd and Pb concentrations.
[107]	Low (*n* = 61) and normal (*n* = 55) quality semen groups	S and B	ICP-MS	Pb, Cd, As, Ba, Hg and U	- Low-quality semen participants had significantly higher Cd and Ba concentrations in semen. - A significant association between low sperm viability and higher blood Cd and Ba, as well as higher seminal Pb, Cd, Ba and U. - U concentrations in semen were associated with increased odd ratios for below-reference progressive sperm motility and normal morphology.
[108]	Infertile men (*n* = 20) and fertile male volunteers as a control group (*n* = 20)	SP and SE	AAS	Se, Pb and Cd	- The infertile group showed lower concentration of Se in SE. - Additionally, the infertile group had higher levels of Cd and Pb in SE and SP. - A significant negative correlation between TOS and Se concentration was found in SE.
[109]	Men attending an *in vitro* fertilization (IVF) treatment (*n* = 30)	SP	ICP-MS	Hg, Cd and Pb	- Higher SP Pb concentrations were associated with lower total motile sperm. - A positive association was found between SP Cd and sperm concentration. - Higher concentrations of Cd in SP had lower probabilities for implantation and pregnancy. - Positive associations were found between SP Hg and embryo quality, implantation, pregnancy and live birth.
[110]	Case subjects (*n* = 30) with oligo-astheno-teratozoospermia and normozoospermic men (*n* = 31) as control subjects, all attending fertility clinics	SP, B and WB	Anodic stripping voltammetry and AAS	Cd, Pb and Hg	- No significant differences found between cases and controls in heavy metal contents in any of the three biological matrices. - A significant positive association was found between the percentage of immotile sperms and SP levels of Pb and Cd. - The 3 heavy metals (Pb, Cd and Hg) were highly correlated among themselves, when compared within the same bodily fluid.
[111]	Men attending an accademic fertility center (*n* = 129)	H	Direct Mercury Analyzer 80	Hg	- Hg was positively associated with normal sperm morphology, concentration, total sperm count, progressive motility and progressive motile sperm count.
[112]	Men (*n* = 47) undergoing semen analysis in an infertility center	S	Cd and Pb were determined by the voltametric method. Mg, Cu and Zn by F-AAS	Cd, Pb, Ni, Fe, Mg, Cu and Zn	- Correlation between Pb and flagellum ball, Cd and large heads, and between Fe and other forms of pathological spermatozoa (teratoid spermatozoa, spiral twisted flagellum, deformation of mitochondrial segment).
[113]	Couples (*n* = 104) from China	SP	ICP-MS	Cr, As, Se, Ni Cd and Pb	- SP trace elements were not associated with sperm quality.
[114]	Men with normozoospermia (*n* = 25) as a control group, oligozoospermia (*n* = 25) and azoospermia (*n* = 25)	SP	ICP-MS	Mg, Ca, Al, Ti, V, Cr, Mn, Fe, Co, Ni, Cu, Zn, Sr, Cd, Sn, Sb, Ba, Hg and Pb	- Cd and Ni showed significant differences between the three groups studied. Ni and Cd levels in the SP were negatively correlated with sperm concentration and motility.
[115]	Men from an infertility clinic (*n* = 349)	U	ICP-MS	As, Cd, Co, Cr, Cu, Fe, Pb, Mn, Mo, Hg, Ni, Se and Zn	- Significant trend for decreased odds ratios (ORs) for lower sperm count at higher Se quartiles. - A significant trend for sperm with abnormal morphology at higher Ni quartiles.
[116]	Infertile men from an infertility clinic (*n* = 207)	U	ICP-MS	As, Cd, Co, Cr, Cu, Fe, Pb, Mn, Mo, Hg, Ni, Se and Zn	- Urinary Hg and Ni were associated, in the comet assay, with higher trends for sperm with DNA damage. - Urinary Mn was associated with a higher trend for sperm with DNA damage.

ART, assisted reproductive techniques; B, blood; BP, blood plasma; WB, whole blood; H, hair; S, semen; SE, serum; SP, seminal plasma; U, urine; AAS, atomic absorption spectroscopy; F-AAS, flame-atomic absorption spectroscopy; ICP-MS, inductively coupled plasma-mass spectrometry; Polarized Zeeman AAS, polarized zeeman atomic absorption spectroscopy; Al, Aluminum; As: Arsenic; Ba, Barium; Ca, Calcium; Cd, Cadmium; Cr, Chromium; Co, Cobalt; Cu, Copper; Fe, Iron; Hg, Mercury; Mg, Magnesium; Mn, Manganese; Mo, Molybdenum; Ni, Nickel; Pb, Lead; Sb, Antimony; Se, Selenium; Sr, Strontium; Ti, Titanium; U, Uranium; V, Vanadium; Zn, Zinc.

**Table 6:** Articles studying the relationship between heavy metals and reproductive outcomes in couples from assisted-reproduction centers.

Ref.	Exposure	Study Groups	Biological Matrix	Detection Method	Metal Profile	Main Findings
[118]	Pre-conception parental exposures	A cohort of couples (*n* = 501), including 235 singletons born to 347 couples	B and U	ICP-MS	Pb, Cd and total Hg in blood. Sb, As, Ba, Be, Cd, Cs, Cr, Co, Cu, Pb, Mn, Mo, Ni, Pt, Se, Te, Tl, Sn, W, U, Zn in urine.	- Shorter gestational age at delivery (GA) was related to a higher paternal urine Ba, W and U. - Birth length (BL) was shorter for higher tertiles of urine paternal U (−1.10 cm). Higher tertiles of paternal (+1.30) blood Hg were associated with longer BL. - At birth, head circumference was lower for higher tertiles of paternal urine U (−0.83 cm) and for higher Mo in boys (−0.57 cm).
[119]	Effect of heavy metals at environmentally relevant concentrations on couple fecundity	Couples (*n* = 501) desiring pregnancy	B	ICP-MS	Cd, Pb and Hg	- For both female Cd and male Pb concentrations, lower fecundability odd ratios (FORs) were seen. - Considering the couples’ exposure, only male concentration of Pb significantly reduced the FOR.
[120]	No	Couples (*n* = 103) who underwent IVF/intracytoplasmic sperm injection (ICSI) treatment in a reproduction center	S	ICP-MS	Cr, Ni, As, Se, Cd and Pb	- The concentration in S of As was significantly associated with a greater embryo quality. - Higher Se levels in the seminal plasma of male partners was related to higher pregnancy (implantation and clinical pregnancy) and live-birth probabilities.
[121]	Evaluation of the role of oxidative stress in lifestyle and environmental factors	Couples (*n* = 253) from an IVF center	B, SE and SP	AAS	Pb, Cd, Cu and Zn	- Embryo transfer and IVF outcome may be improved due to higher Zn levels in SP and SE.

IVF, *in vitro* fertilization; B, blood; S, semen; SE, serum; U, urine; AAS, atomic absorption spectroscopy; ICP-MS, inductively coupled plasma-mass spectrometry; As, Arsenic; Ba, Barium; Be. Beryllium; Cd, Cadmium; Co, Cobalt; Cr, Chromium; Cs, Cesium; Cu, Copper; Hg, Mercury; Mn, Manganese; Mo, Molybdenum; Ni, Nickel; Pb, Lead; Pt, Platinum; Sb, Antimony; Sc, Scandium; Se, Selenium; Sn, Tin; Te, Tellurium; Tl, Thallium; U, Uranium; W, Wolframium; Zn, Zinc.

## Supplementary Materials

The following are available online at: https://www.mdpi.com/article/10.3390/antiox10091473/s1, Table S1: Reviews included in the database after application of the inclusion and exclusion criteria, Table S2: Clinical trials included in the database after application of the inclusion and exclusion criteria, Table S3: Articles included in the database after application of the inclusion and exclusion criteria that studied the relationship between heavy metals and human male fertility.
