# Supplementary material for: Impact of Heavy Metals on Human Male Fertility—An Overview
Source: Antioxidants (Basel). 2021 Sep 15;10(9):1473. doi: 10.3390/antiox10091473 (PMC8468047; doi:10.3390/antiox10091473)
Supplement: Supplementary file 1 [file antioxidants-10-01473-s001.zip › antioxidants-1360402-supplementary.pdf]

Supplemental information

Table S1. Tittle, author and year of the 52 reviews analyzed in this study that reviewed the relationship between heavy metals and human male fertility.

Reference	Tittle
**Abarikwu 2013**	Causes and risk factors for male-factor infertility in Nigeria
**Ashiru and Odusanya 2009**	Fertility and occupational hazards: Review of the literature
**Balabanic and Klemencic 2014**	Diet containing endocrine-disruptors and reproductive health
**Bjørklund et al. 2019**	Mercury exposure and its effects on fertility and pregnancy outcome
**Bonde 2010**	Male reproductive organs are at risk from environmental hazards
**Dai et al. 2015**	The hazardous effects of tobacco smoking on male fertility
**de Angelis et al. 2017**	The environment and male reproduction: The effect of cadmium exposure on reproductive function and its implication in fertility
**DeCelis et al. 1996**	Toxicology of male reproduction in animals and humans
**Droller 1996**	Environment and the genitourinary tract
**Fallah et al. 2018**	Zinc is an essential element for male fertility: A review of zn roles in men's health, germination, sperm quality, and fertilization
**Figa-Talamanca et al. 2001**	Occupational exposures to metals, solvents and pesticides: Recent evidence on male reproductive effects and biological markers.
**Giudice 2006**	Infertility and the environment: The medical context
**Grigore and Indrei, 2001**	The role of heat shock proteins in reproduction
**Hales and Robaire 2001**	Review - paternal exposure to drugs and environmental chemicals: Effects on progeny outcome
**Henriques et al. 2019**	Exposure to mercury and human reproductive health: A systematic review
**Hruska et al. 2000**	Environmental factors in infertility
**Ilieva et al. 2020**	Toxic effects of heavy metals (lead and cadmium) on sperm quality and male fertility
**Jenardhanana et a.l 2016**	Effect of environmental contaminants on spermatogenesis.
**Jensen et al. 2006**	The influence of occupational exposure on male reproductive function
**Kaminski et al. 2020**	External and genetic conditions determining male infertility
**Kumar 2004**	Occupational exposure associated with reproductive dysfunction
**Kumar 2018**	Occupational and environmental exposure to lead and reproductive health impairment: An overview

**Kumar and Sharma 2019**	Cadmium toxicity: Effects on human reproduction and fertility
**Lavranos et al. 2012**	Investigating ROS sources in male infertility: A common end for numerous pathways
**Lovakovic 2020**	Cadmium, arsenic, and lead: Elements affecting male reproductive health
**Ma et al. 2016**	Effects of environmental contaminants on fertility and reproductive health.
**Manfo et al. 2014**	Effect of environmental contaminants on mammalian testis.
**Martynowicz et al. 2005**	Effects of cadmium on testis function.
**Marzec-Wroblewska et al. 2012**	Influence of chemical elements on mammalian spermatozoa
**Najafi et al. 2015**	Air pollution and quality of sperm: A meta-analysis.
**Neuer et al. 2000**	The role of heat shock proteins in reproduction
**Ogorek et al. 2017**	Role of copper in the process of spermatogenesis
**Pieczynska and Grajeta 2015**	The role of selenium in human conception and pregnancy
**Plunk and Richards 2020**	Endocrine-disrupting air pollutants and their effects on the hypothalamus-pituitary-gonadal axis
**Ramos-Trevino et al. 2018**	Toxic effect of cadmium, lead, and arsenic on the Sertoli cell: Mechanisms of damage involved.
**Reis et al. 2015**	Sertoli cell as a model in male reproductive toxicology: Advantages and disadvantages
**Rezk and Sikka 2011**	Developmental and reproductive disorders: Role of endocrine disruptors in testicular toxicity
**Rier 2008**	Environmental immune disruption: A comorbidity factor for reproduction?
**Rogers 2008**	Tobacco and pregnancy: Overview of exposures and effects
**Sengupta et al. 2017**	Evidence for decreasing sperm count in African population from 1965 to 2015
**Sheiner et al. 2003**	Effect of occupational exposures on male fertility: Literature review
**Sheweita et al. 2005**	Mechanisms of male infertility: Role of antioxidants
**Siu et al. 2009**	Cadmium-induced testicular injury
**Skolarczyk et al. 2018**	The impact of cadmium on male infertility
**Takeda et al. 2004**	Endocrine-disrupting activity of chemicals in diesel exhaust and diesel exhaust particles
**Thompson and Bannigan, 2008**	Cadmium: Toxic effects on the reproductive system and the embryo
**Vigeh et al. 2011**	How does lead induce male infertility?
**Wan et al. 2013**	Targeting testis-specific proteins to inhibit spermatogenesis: Lesson from endocrine disrupting chemicals.
**Webb et al. 2014**	Developmental and reproductive effects of chemicals associated with unconventional oil and natural gas operations

**Wirth and Mijal, 2010**	Adverse effects of low-level heavy metal exposure on male reproductive function
**Zenzes 2000**	Smoking and reproduction: Gene damage to human gametes and embryos
**Zhang et al. 2019**	Relationship between cadmium content in semen and male infertility: A meta-analysis

Table S2. Tittle, author and year of 2 clinical trials analyzed in this study that studied the relationship between heavy metals and human male fertility.

Author	Tittle
**Alexander et al. 1998**	Interaction of blood lead and delta-aminolevulinic acid dehydratase genotype on markers of heme synthesis and sperm production in lead smelter workers
**Ali et al. 2017**	Mapping fifteen trace elements in human seminal plasma and sperm DNA

Table S3. Tittle, author and year of the 90 articles analyzed in this study that studied the relationship between heavy metals and human male fertility.

Author	Tittle
**Apostoli et al. 1999**	Critical aspects of male fertility in the assessment of exposure to lead
**Araya et al. 2003**	Human spermatozoa motility analysis in a ringer's solution containing cupric ions
**Balabanic et al. 2011**	Negative impact of endocrine-disrupting compounds on human reproductive health
**Benoff et al. 2000**	Metal ions and human sperm mannose receptors
**Bloom et al. 2015**	Birth outcomes and background exposures to select elements, the longitudinal investigation of fertility and the environment (LIFE)
**Chen et al. 2015**	Rare earths exposure and male infertility: The injury mechanism study of rare earths on male mice and human sperm.
**Comhaire et al. 2007**	Sperm quality, birth rates and the environment in Flanders (Belgium)
**Dandevi et al. 2003**	Semen quality of Indian welders occupationally exposed to nickel and chromium

**De Fleurian et al. 2009**	Occupational exposures obtained by questionnaire in clinical practice and their association with semen quality
**Dickman and Leung 1998**	Mercury and organochlorine exposure from fish consumption in Hong Kong.
**Dickman et al. 1998**	Hong Kong male subfertility links to mercury in human hair and fish.
**Elsamanoudy et al. 2014**	Possible effects of metallosis on spermatozoal apoptotic genes expression in individuals with intramedullary nailing prosthesis
**Evans 2011**	Endocrine disruptors
**Fatima et al. 2015**	Association of blood and semen lead and zinc level with semen parameter in the male partner of infertile couple
**Garcia-Herrero et al. 2011**	Differential transcriptomic profile in spermatozoa achieving pregnancy or not via ICSI
**Gerhard et al. 1998**	Heavy metals and fertility
**Ghaffari and Motlagh 2011**	In vitro effect of lead, silver, tin, mercury, indium and bismuth on human sperm creatine kinase activity: A presumable mechanism for men infertility
**Ghiasvand et al. 2020**	The association between animal flesh foods consumption and semen parameters among infertile Iranian men: A cross-sectional study
**Hanf et al. 1996**	Mercury in urine and ejaculate in husbands of barren couples
**Hardneck et al. 2018**	Quantitative assessment of heavy metal effects on sperm function using computer-aided sperm analysis and cytotoxicity assays.
**Hassan et al. 2019**	The protective effect of epigallocatechin-3-gallate on testicular oxidative stress in lead-induced toxicity mediated by Cyp19 gene / estradiol level
**He et al. 2016**	Lead inhibits human sperm functions by reducing the levels of intracellular calcium, cAMP, and tyrosine phosphorylation
**He et al. 2020**	Heavy metal exposure, oxidative stress and semen quality: Exploring associations and mediation effects in reproductive-aged men
**Huang et al. 2001**	In vitro effects of metal ions (Fe2+, Mn2+, Pb2+) on sperm motility and lipid peroxidation in human semen
**Hunter 2011**	The cost of living longer fertility trades with immunity and life expectancy
**Inhorn et al. 2008**	Occupational and environmental exposures to heavy metals: Risk factors for male infertility in Lebanon?
**Irgens et al. 1999**	The effect of male occupational exposure in infertile couples in Norway.
**Jeng et al. 2015**	Role of low exposure to metals as male reproductive toxicants.
**Kahraman et al. 2012**	The effect of blood and seminal plasma heavy metal and trace element levels on sperm quality
**Keck et al. 1995**	Lack of correlation between cadmium in seminal plasma and fertility status of nonexposed individuals and 2 cadmium-exposed patients
**Kim et al. 2014**	Toxic metals in seminal plasma and in vitro fertilization (IVF) outcomes
**Kislitskaya et al. 2015**	Disturbance of antioxidant enzymes and purine metabolism in the ejaculate of men living in disadvantaged areas of Kyzylorda region.
**Kumar et al. 2000**	Chlorinated pesticides and heavy metals in human semen

**Kumar et al. 2018**	Role of environmental factors & oxidative stress with respect to in vitro fertilization outcome
**Kuo et al. 1997**	Semen quality in workers with long-term lead exposure: A preliminary study in Taiwan
**Leite et al. 2015**	Environmentally realistic doses of cadmium as a possible etiologic agent for idiopathic pathologies
**Lettieri et al. 2020**	Discovery of the involvement in DNA oxidative damage of human sperm nuclear basic proteins of healthy young men living in polluted areas
**Lettieriet al. 2020**	Molecular alterations in spermatozoa of a family case living in the land of fires-A first look at possible transgenerational effects of pollutants
**Li et al. 2008**	Are serum zinc and copper levels related to semen quality?
**Li et al. 2015**	Biomonitoring of blood heavy metals and reproductive hormone level related to low semen quality
**Li et al. 2014**	Cadmium, chromium, and copper concentration plus semen-quality in environmental pollution site, china
**Li et al. 2012**	Effects of manganese on routine semen quality parameters: Results from a population-based study in china
**Li et al. 2016**	Association between environmental exposure to cadmium and human semen quality.
**Lin et al. 1996**	Fertility rates among lead workers and professional bus drivers: A comparative study
**Louis et al. 2012**	Heavy metals and couple fecundity, the LIFE study
**Marchiani et al. 2019**	Acute effects on human sperm exposed in vitro to cadmium chloride and diisobutyl phthalate.
**Mendiola et al. 2011**	Relationships between heavy metal concentrations in three different body fluids and male reproductive parameters: A pilot study
**Minguez-Alarcon et al. 2018**	Hair mercury (hg) levels, fish consumption and semen parameters among men attending a fertility center.
**Mitra et al. 2020**	Lead and cadmium exposure induces male reproductive dysfunction by modulating the expression profiles of apoptotic and survival signal proteins in tea -garden workers
**Monsees et al. 2000**	Sertoli cells as a target for reproductive hazards
**Moran-Martinez et al. 2013**	Chronic environmental exposure to lead affects semen quality in a Mexican men population
**Mukhopadhyay et al. 2010**	CASA-based sperm kinematics of environmental risk factor-exposed human semen samples designated as normozoospermic in conventional analysis
**Murado et al. 2014**	Oversimplification and overstandardization in biological methods: Sperm bioassays in ecotoxicology as a case of study and a proposal for their reformulation.
**Naha and Chowdhury 2006**	Inorganic lead exposure in battery and paint factory: Effect on human sperm structure and functional activity
**Naha et al. 2005**	Structural alteration of spermatozoa in the persons employed in lead acid battery factor
**Nath et al. 2010**	Heavy metal contaminants and male reproductive health

**Nivsarkar et al. 1998**	A regulatory role of sulfhydryl groups in modulation of sperm membrane conformation by heavy metals: Sulfhydryl groups as markers for infertility assessment
**Nykolaichuk et al. 2020**	Impact of environmental factors on male reproductive health
**Pant et al.2013**	Semen quality of environmentally exposed human population: The toxicological consequence.
**Pant et al. 2003**	Lead and cadmium concentration in the seminal plasma of men in the general population: Correlation with sperm quality.
**Pavlova and Atanassova 2018**	Impact of cadmium on male fertility
**Petrelli et al. 2001**	Occupational exposure and male fertility. results of an Italian multicenter study in an exposed population].
**Quansah and Jaakkola 2009**	Paternal and maternal exposure to welding fumes and metal dusts or fumes and adverse pregnancy outcomes
**Rao et al. 2015**	Evaluation of semen quality in 1808 university students, from Wuhan, central china
**Riaz et al. 2016**	Impact of reactive oxygen species on antioxidant capacity of male reproductive system.
**Robert et al. 1997**	Characterization of prostate-specific antigen proteolytic activity on its major physiological substrate, the sperm motility inhibitor precursor semenogelin I
**Sengupta et al. 2013**	Human sperm and other seminal constituents in male infertile patients from arsenic and cadmium rich areas of southern assam.
**Shi et al. 2020**	Interaction effect of polycyclic aromatic hydrocarbon metabolites and cadmium on semen quality in nonsmokers
**Skandhan et al. 2017**	Level of copper in human split ejaculate
**Slivkova et al. 2009**	Concentration of trace elements in human semen and relation to spermatozoa quality.
**Snijder et al. 2011**	Occupational exposure to endocrine disruptors and time to pregnancy among couples in a large birth cohort study: The generation R study
**Sobolev et al. 2018**	Biological role of selenium in the organism of animals and humans
**Stassen et al. 2012**	Metal exposure and reproductive disorders in indigenous communities living along the Pilcomayo river, Bolivia
**Stoltenberg et al. 1997**	Histochemical demonstration of zinc ions in human epididymis using autometallography.
**Stoltenberg et al. 1997**	Histochemical demonstration of zinc ions in ejaculated human semen
**Sukhn et al. 2018**	Associations of semen quality with non-essential heavy metals in blood and seminal fluid: Data from the environment and male infertility (EMI) study in Lebanon.
**Sun et al. 2017**	Heavy metal level in human semen with different fertility: A meta-analysis.
**Telisman et al. 2007**	Reproductive toxicity of low-level lead exposure in men.
**Telisman et al. 2000**	Semen quality and reproductive endocrine function in relation to biomarkers of lead, cadmium, zinc, and copper in men

**Wang et al. 2017**	Associations between urinary polycyclic aromatic hydrocarbon metabolites and serum testosterone in US adult males: National health and nutrition examination survey 2011-2012.
**Wang et al. 2016**	Variability of metal levels in spot, first morning, and 24-hour urine samples over a 3-month period in healthy adult Chinese men
**Wang et al. 2017**	Relationships between seminal plasma metals/metalloids and semen quality, sperm apoptosis and DNA integrity
**Wijesekara et al. 2015**	Environmental and occupational exposures as a cause of male infertility
**Wu et al. 2012**	Lead level in seminal plasma may affect semen quality for men without occupational exposure to lead
**Wu et al. 2020**	Associations of toxic and essential trace elements in serum, follicular fluid, and seminal plasma with in vitro fertilization outcomes
**Wulff et al. 1997**	Infertility in an industrial setting - A population-based study from northern Sweden.
**Yoshida et al. 2008**	Physiological roles of semenogelin I and zinc in sperm motility and semen coagulation on ejaculation in humans
**Zafar et al. 2015**	Toxic metals signature in the human seminal plasma of Pakistani population and their potential role in male infertility
**Zeng et al. 2015**	Urinary metal concentrations in relation to semen quality: A cross-sectional study in china
**Zhou et al. 2016**	Evaluation of urinary metal concentrations and sperm DNA damage in infertile men from an infertility clinic
